# Supplementary material for: Indexing and partitioning the spatial linear model for large data sets
Source: PLoS One. 2023 Nov 1;18(11):e0291906. doi: 10.1371/journal.pone.0291906 (PMC10619847; doi:10.1371/journal.pone.0291906)
Supplement: Attachment — Submitted filename: rebuttalLetter.pdf [file pone.0291906.s002.pdf]

MARINE MAMMAL
LABORATORY

Jay M. Ver Hoef, Statistician

NOAA-NMFS Alaska Fisheries Science Center
7600 Sand Point Way NE
Seattle, WA 98115
Phone: (907) 456-4321
E-mail: jay.verhoef@noaa.gov
URL: https://sites.google.com/site/jayverhoef/

June 15, 2023

Professor Mohamed R. Abonazel
PLOS ONE Academic Editor

Dear Professor Abonazel,

Thank you for allowing a re-submission of our manuscript, "Indexing and Partitioning the Spatial Linear Model for Large Data Sets," by myself, Michael Dumelle, Matt Higham, Erin Peterson, and Daniel Isaak. In this letter we address all of the issues provided by yourself and the reviewer. A marked-up copy of the revision is included, and well as an unmarked revision. We appreciate the time and comments from you and the reviewer.

Response to Editor Abonazel

- 1. We have checked again that the manuscript meets the PLOS ONE style, using the documents that you provided, and we have used the PLOS ONE Latex template. The revised manuscripts meets these style guidelines.
- 2. We have created a Github repository for an R package with all data and code which can be found here, and has been added to the Data and Software Availability section

  `https://github.com/jayverhoef/SPIN`
- 3. We have removed any funding statements and revised the Acknowledgments. We would like to keep the funding statement for the online submission as you suggest, "JVH: The project received financial support through Interagency Agreement DW-13-92434601-0 from the U.S. Environmental Protection Agency (EPA), and through Interagency Agreement 81603 from the Bonneville Power Administration (BPA), with the National Marine Fisheries Service, NOAA. The funders had no role in study design, data collection and analysis, decision to publish, or preparation of the manuscript."
- 4. Figures 1 and 5 are **not** copyrighted maps. These are original figures that **we** created in the software package `R` using the `sp` package from freely available raw data for streams and state boundaries. No permissions are required.

Response to Reviewer 1

Thank you very much for this review. Below, we copy the suggestion as text in a light brown color, and provide our response in black text.

- 1. There are too many variables (parameters) in this article, I suggest to add a table of acronym to define each variable separately.
  Thank you very much for this suggestion. It is now Table 1, and it has made the paper easier to read.
- 2. What is Eq (1)? You should write Equation (1).
  The Latex template that we used states explicitly 'Use "Eq" instead of "Equation" for equation citations.' We also looked at recent PLOS ONE publications, and most of them used the abbreviation "Eq". We used

"Eq" as recommended by the template.

- 3. $\mathbf{\Sigma}$ is the standard notation for sum, but you used for different concept. You should avoid using standard notation for different concepts.
  $\mathbf{\Sigma}$ is the usual symbol for a covariance matrix in the statistical literature, but we changed the covariance matrix to $\mathbf{V}$ to comply with the reviewer's request.
- 4. In the literature review, you used 13-16, 17-19, 20-22, 27-29. You should explain these separately.
  We are not exactly sure what is meant here. We used several citations to show that a single concept has been used repeatedly in the literature, and hence a range of citation numbers. On the other hand, if the reviewer is suggesting using a format like 13, 14, 15, 16, then the 13-16 format is how the PLOS ONE Latex template chooses to format multiple citations, and it is out of our control. No changes were made.
- 5. Figure 1 is not on the place of Figure 1, you should place your figures over there where you claimed.
  Again, we are using the PLOS ONE Latex template and the figures float in the document. All figures are reasonably close to their first mention, and we do not have control on absolute placement.
- 6. Where did you get the methodology? Is it standard or your own novelty? You did not cited a single reference there.
  In the Introduction, at the end of the Spatial Methods for Big Data section, we state "A blocked structure for the covariance matrix based on spatially-compact groupings was proposed by [54], who then formulated a hybrid likelihood based on blocks of different sizes. The method that we feature is most similar to [54], but we show that there is no need for a hybrid likelihood, and that our approach is different than composite likelihood." Our Methods section goes on to show exactly how our method is different. For example, we state, "Partitioning the covariance matrix is most closely related to the idea of quasi-likelihood [62], composite likelihood [45] and divide and conquer [63]. However, for REML, they are not exactly equivalent," and then we go on to show how our method is different. Our most important novelty is given after Equation (13), where we state "Partitioning of the spatial linear model allows computation from Eq (11), but then going back to the full model for developing Eq (13), which is a new result. This can be contrasted to the approaches for variance estimation of fixed effects using pseudo likelihood, composite likelihood, and divide and conquer found in the earlier literature review." For point prediction, we give original credit in the following sentence, "We recommend point predictions based on local data instead, which is an old idea, e.g. [43], and has already been implemented in software for some time, e.g. [10]." We then show that our method is slightly different, especially as a "local predictor lacks model coherency, as discussed in the literature review section." (The beginning of the 3rd paragraph in the Point Prediction section). The whole collection of inferences, from estimating covariance parameters and fixed effects, to making point and block predictions, is a new methodology in its entirety (which we called SPIN), and we think that we have documented it properly. However, to make it clearer, we have added an **Objectives** section at the end of the Introduction, which defines the acronym SPIN right away, and gives a road-map to what we cover in the Methods section.
- 7. There are too many short forms in this article. I suggest again to create a new table to define each short form separately. See Page 12/26.
  Thank you for this suggestion. We have included it (now, Table 2), and it helps make the article more readable.
- 8. Tables are not on the place of tables, you should place your tables over there where you claimed.
  As we explained about figures, tables also "float" when using Latex. We have placed the tables immediately after their first mention in the Latex document, and specific placement depends on Latex processing.
- 9. The literature about Partitioning is very limited, I recommend to add more detail about graph theory. I recommend to add the following basics, Notes on the Localization of Generalized Hexagonal Cellular Networks, Mathematics. DOI: 10.3390/math11040844. Verification of some topological indices of Y-junction based nanostructures by M-polynomials. Journal of Mathematics. DOI: 10.1155/2022/8238651. Sharp bounds on partition dimension of hexagonal Mobius ladder. Journal of King Saud University-Science, Dec. 2021. DOI:10.1016/j.jksus.2021.101779. Metric-based resolvability of polycyclic aromatic hydrocarbons. European Physical Journal Plus. DOI:10.1140/epjp/s13360-021-01399-8
  We appreciate this list of articles, but our methods are not based on graph theory in any way. We are modeling spatially-continuous surfaces. We looked at these articles, which are interesting for partitioning of graphical models, but we do not see how they are relevant for our article. We are partitioning covariance

matrices representing continuous surfaces, which are not graphical models.

- 10. In page 17/26, line 496, what is covariance matrice? A typo.
  Thank you, it has been corrected.
- 11. There are many typos in this article, you should recheck this article.
  We have checked the manuscript for further typos. The changes can be found in the markup copy.
- 12. References are not in the same pattern see 59 and 60 (page section).
  All references are formatted using the PLOS ONE Latex template. The reason that the patterns are different is because one is a journal article, and the other part of a Proceedings.
- 13. See the difference in journal name of ref 62 with others.
  Same comment as above.
- 14. Figures are very blur unable to understand, kindly revise the quality of pictures.
  Thank you for this suggestion. Figures 1, 2, and 5 have hundreds of thousands of points and lines, so high resolution images make file sizes very large. For the first submission, we used PNG format to keep the file size smaller. In this revision, we have included the full EPS format as required by PLOS ONE.
- 15. Try to write the article in understandable way, you are teaching newcomers not only publishing articles.
  We think that the changes to the manuscript that the reviewer has suggested have added greatly to its clarity and accessibility to a broader audience. However, we do not know how to respond to this comment because it lacks specific suggestions. We are not trying to teach newcomers (that is not the only goal, nor even the primary one, of PLOS ONE). Rather, we are trying to make a novel improvement to geostatistical methods for very large spatial data, and given that, we want as broad an audience as possible for reading about our method while maintaining statistical rigor. To achieve that rigor, we must use statistical concepts and vocabulary. The set of authors who have contributed to this article include statisticians, spatial scientists, and fish biologists. Together, we have a lot of experience co-publishing in both the statistical and environmental literature, and we feel the paper meets the standards for good writing.
- 16. Elaborate each section in an understandable way, make section and enumerate them.
  Same comment as above, with the addition that PLOS ONE does not allow the enumeration of sections. We have followed the formatting guidelines.

Sincerely,

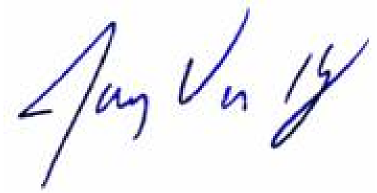

Jay M. Ver Hoef, Ph.D.
