# Supplementary material for: Indexing and partitioning the spatial linear model for large data sets
Source: PLoS One. 2023 Nov 1;18(11):e0291906. doi: 10.1371/journal.pone.0291906 (PMC10619847; doi:10.1371/journal.pone.0291906)
Supplement: Attachment — Submitted filename: rebuttalLetter2.pdf [file pone.0291906.s003.pdf]

MARINE MAMMAL
LABORATORY

Jay M. Ver Hoef, Statistician

NOAA-NMFS Alaska Fisheries Science Center
7600 Sand Point Way NE
Seattle, WA 98115
Phone: (907) 456-4321
E-mail: jay.verhoef@noaa.gov
URL: https://sites.google.com/site/jayverhoef/

August 24, 2023

Professor Mohamed R. Abonazel
PLOS ONE Academic Editor

Dear Professor Abonazel,

Thank you for allowing a second re-submission of our manuscript, "Indexing and Partitioning the Spatial Linear Model for Large Data Sets," by myself, Michael Dumelle, Matt Higham, Erin Peterson, and Daniel Isaak. In this letter we address all of the issues provided by yourself and both reviewers. A marked-up copy of this second revision, showing the changes from the first revision, is included, as well as an unmarked revision. We appreciate the time and comments from you and both reviewers.

Response to Reviewer 1

You may accept this article from my side. Authors addressed all the suggested comments from my side. It is quite to accept this article.

Thank you very much for your helpful comments in the previous review.

Response to Reviewer 2

The authors present an approximate inference method for large spatial datasets by indexing and partitioning the data into blocks and taking these blocks to be independent, thus reducing the computational load. While this approach is not new, the authors present enough variations and innovations to differentiate it from the already published methods. The authors provide simulated examples where the fixed effects in the model were estimated with fair accuracy. The method is applied to a real dataset with success.

The article is written well and is easily readable for people who engage in practicing spatial statistical methods. However, I have the following comments about the work:

Thank you very much for your comments, especially the clarification on RMSPE. We are unsure if you were working from the original manuscript or the previous revision. At any rate, we hope that our responses below will be satisfactory.

- 1. Is the primary objective of this method to efficiently estimate the covariate effects? You do not present covariance parameter estimation results anywhere.
  Thank you for this comment. Our primary objectives, as we stated in the Objectives section, are to use SPIN to quickly and validly 1) estimate covariance parameters, 2) estimate fixed effects for covariates, and 3) prediction (both point and block). To make covariance parameter estimation clearer, we added the section header for "Estimation of Covariance Parameters." All other inferences require the covariance parameter estimates in order to parameterize the covariance matrix (with plug-in estimates), and this is the slowest computation because it is iterative for the inverse of the covariance matrix, so this is where SPIN is most important. However, most users are mainly interested in estimating fixed effects and prediction, and

our most novel contribution is how to correctly estimate the variances of the estimated fixed effects. We hope the slightly altered structure and few sentences help to make this clearer.

- 2. I am skeptical about the spatial prediction performance of the method. As per the described data generation method, the magnitude of the response variable Y would be around 40 at most. From Tables 3 and 4, the RMSPE is of similar magnitude. This indicates rather poor performance and since you do not compare these to any other method, it is hard to say if the method is working well or not. Please correct me about the magnitude of Y, if I am wrong.
  Thank you very much for this comment! After checking the code, we realized that we forgot to take the square roots when computing RMSPE (thus, is was MSPE.) We have corrected Tables 3, 4, and 6 for the RMSPE columns.
- 3. The RMSPE in Table 7 is surprisingly low compared to the numbers in Tables 3, 4, and 6. Are the data generated similarly, with the same scale of Y in both cases. If so, what lead to such large improvements?
  Again, thank you very much for this comment! Again, the RMSPE column was missing the square root, and we standardized the errors prior to adding fixed effects. We have clarified this in the text describing the simulation, and corrected the Table.
- 4. This is a minor comment, but the spatial range parameter being bigger than 1 (or even 0.5) for a field of size unit square is unrealistic. But for investigating purposes, it is fine.
  Although Fig. 2 used a range of 2 to make the spatial patterning very apparent, we used a range parameter of 0.5 for a spherical model for all simulations.
- 5. Eq (19) has a typo, it should be Vj in the second term and not Vi.
  Thank you, we corrected it.

Sincerely,

Jay M. Ver Hoef, Ph.D.
