# Supplementary material for: Indexing and partitioning the spatial linear model for large data sets
Source: PLoS One. 2023 Nov 1;18(11):e0291906. doi: 10.1371/journal.pone.0291906 (PMC10619847; doi:10.1371/journal.pone.0291906)
Supplement: Attachment — Submitted filename: PONE-D-23-03446_reviewers.docx [file pone.0291906.s001.docx]

Indexing and Partitioning the Spatial Linear Model for Large Data Sets

Few major comments are attached for the improvement of the article.

1. There are too many variables (parameters) in this article, I suggest to add a table of acronym to define each variable separately.
2. What is Eq (1)? You should write Equation (1).
3. Σ is the standard notation for sum, but you used for different concept. You should avoid using standard notation for different concepts.
4. In the literature review, you used 13-16, 17-19, 20-22, 27-29. You should explain these separately.
5. Figure 1 is not on the place of Figure 1, you should place your figures over there where you claimed.
6. Where did you get the methodology? Is it standard or your own novelty? You did not cited a single reference there.
7. There are too many short forms in this article. I suggest again to create a new table to define each short form separately. See Page 12/26.
8. Tables are not on the place of tables, you should place your tables over there where you claimed.
9. The literature about Partitioning is very limited, I recommend to add more detail about graph theory. I recommend to add the following basics, Notes on the Localization of Generalized Hexagonal Cellular Networks, Mathematics. DOI: 10.3390/math11040844. Verification of some topological indices of Y-junction based nanostructures by M-polynomials. Journal of Mathematics. DOI: 10.1155/2022/8238651. Sharp bounds on partition dimension of hexagonal Mobius ladder. Journal of King Saud University-Science, Dec. 2021. DOI:10.1016/j.jksus.2021.101779. Metric-based resolvability of polycyclic aromatic hydrocarbons. European Physical Journal Plus. DOI:10.1140/epjp/s13360-021-01399-8
10. In page 17/26, line 496, what is covariance matrice? A typo.
11. There are many typos in this article, you should recheck this article.
12. References are not in the same pattern see 59 and 60 (page section).
13. See the difference in journal name of ref 62 with others.
14. Figures are very blur unable to understand, kindly revise the quality of pictures.
15. Try to write the article in understandable way, you are teaching newcomers not only publishing articles.
16. Elaborate each section in an understandable way, make section and enumerate them.
